# 6-Paradol and 6-Shogaol, the Pungent Compounds of Ginger, Promote Glucose Utilization in Adipocytes and Myotubes, and 6-Paradol Reduces Blood Glucose in High-Fat Diet-Fed Mice

**DOI:** 10.3390/ijms18010168

**Published:** 2017-01-17

**Authors:** Chien-Kei Wei, Yi-Hong Tsai, Michal Korinek, Pei-Hsuan Hung, Mohamed El-Shazly, Yuan-Bin Cheng, Yang-Chang Wu, Tusty-Jiuan Hsieh, Fang-Rong Chang

**Affiliations:** 1 Graduate Institute of Natural Products, Kaohsiung Medical University, Kaohsiung 807, Taiwan; weichankai@yahoo.com.tw (C.-K.W.); lyph0719@hotmail.com (Y.-H.T.); mickorinek@hotmail.com (M.K.); mohamed.elshazly@pharm.asu.edu.eg (M.E.-S.); jmb@kmu.edu.tw (Y.-B.C.); yachwu@mail.cmu.edu.tw (Y.-C.W.); 2 Graduate Institute of Medicine, College of Medicine, Kaohsiung Medical University, Kaohsiung 807, Taiwan; vean1990@hotmail.com; 3 Department of Pharmacognosy and Natural Products Chemistry, Faculty of Pharmacy, Ain-Shams University, Cairo 11566, Egypt; 4 School of Pharmacy, College of Pharmacy, China Medical University, Taichung 404, Taiwan; 5 Chinese Medicine Research and Development Center, China Medical University Hospital, Taichung 404, Taiwan; 6 Center for Molecular Medicine, China Medical University Hospital, Taichung 404, Taiwan; 7 Department of Marine Biotechnology and Resources, College of Marine Sciences, National Sun Yat-sen University, Kaohsiung 804, Taiwan; 8 Lipid Science and Aging Research Center, Kaohsiung Medical University, Kaohsiung 807, Taiwan; 9 Research Center for Environmental Medicine, Kaohsiung Medical University, Kaohsiung 807, Taiwan

**Keywords:** 6-paradol, shogaols, 3T3-L1 adipocytes, C2C12 myotubes, high-fat diet-fed mice, diabetes mellitus

## Abstract

The anti-diabetic activity of ginger powder (*Zingiber officinale*) has been recently promoted, with the recommendation to be included as one of the dietary supplements for diabetic patients. However, previous studies presented different results, which may be caused by degradation and metabolic changes of ginger components, gingerols, shogaols and paradols. Therefore, we prepared 10 ginger active components, namely 6-, 8-, 10-paradols, 6-, 8-, 10-shogaols, 6-, 8-, 10-gingerols and zingerone, and evaluated their anti-hyperglycemic activity. Among the tested compounds, 6-paradol and 6-shogaol showed potent activity in stimulating glucose utilization by 3T3-L1 adipocytes and C2C12 myotubes. The effects were attributed to the increase in 5′ adenosine monophosphate-activated protein kinase (AMPK) phosphorylation in 3T3-L1 adipocytes. 6-Paradol, the major metabolite of 6-shogaol, was utilized in an in vivo assay and significantly reduced blood glucose, cholesterol and body weight in high-fat diet-fed mice.

## 1. Introduction

Ginger (*Zingiber officinale*) is one of the oldest domesticated spices in human history. It is commonly used as a food additive (spice) and as a key component in traditional herbal medicine. The health-enhancing potential of ginger has been intensively explored, and it is considered to be safe as an herbal supplement by different regulatory authorities [1,2,3]. It was reported that ginger could treat a myriad of ailments, including flu-related symptoms and some digestive problems, such as constipation, vomiting and ulcer [4]. Ginger also possesses anthelmintic, anti-bacterial and anti-viral activities [5,6,7]. Moreover, ginger was found to be active against inflammatory, allergic, degenerative, cardiovascular and metabolic disorders [8,9,10].

Diabetes mellitus is one of the most devastating metabolic disorders and one of the leading causes of death worldwide [11]. Several classes of oral anti-diabetic agents have been developed in the second half of the last century targeting this ailment. These main classes include agents that improve insulin sensitivity (thiazolidinediones), stimulate insulin secretion (sulfonylureas and rapid-acting secretagogues), reduce hepatic glucose production (biguanides) or delay digestion and absorption of intestinal carbohydrates (alpha-glucosidase inhibitors) [12,13]. Despite the relative success of these compounds in controlling diabetic mellitus and its complications, the long-term use of these agents is usually accompanied with several undesirable side effects, such as low blood glucose, weight gain, stomachache, diarrhea, anemia or bone fracture in women [14].

Several animal studies and clinical trials examined the effectiveness of ginger as an anti-diabetic agent. One study indicated that consuming 3.0 g/day of dry ginger powder for 30 consecutive days led to a significant reduction in blood glucose, triglycerides, total cholesterol, low-density lipoprotein (LDL) and very low-density lipoprotein (VLDL) in diabetic patients [15]. However, other studies suggested that ginger consumption had an insignificant effect on blood glucose and blood lipids [16,17]. This discrepancy was attributed to the use of unstandardized ginger powder with different concentrations of the active constituents in each study [18]. Therefore, in order to introduce ginger as a potential anti-diabetic agent, it is necessary to evaluate the anti-diabetic, as well as the anti-hyperglycemic and anti-hyperlipidemic effects of its major active constituents [19].

Recent reports suggested that gingerols, the major pungent components of ginger oleoresin, are responsible for the pharmacological properties of ginger [19,20]. These reports provided preliminary data on the activity of gingerols in maintaining glucose homeostasis. In previous studies, gingerols significantly promoted glucose uptake in adipocytes [19] and skeletal muscle cells [21] through the increase in GLUT4 expression on the cell membrane [22] and activation of the AMP-activated protein kinase (AMPK) pathway [23]. Gingerols also showed anti-hyperglycemic and anti-hyperlipidemic effects in type 2 diabetic mice [23]. Ginger oleoresin is composed, in addition to gingerols, of other pungent non-volatile components, including paradols, shogaols and zingerone. These components possess closely-related structures, and they are considered as a homologous series of phenols with different unbranched alkyl chains [24]. Recent studies have indicated that ginger oleoresin components are unstable [5]. Gingerols, the major component of ginger, is converted mainly to zingerone and shogaols due to drying, prolonged storage or cooking [5,25]. The shogaols present in ginger are then partly transformed to paradols upon cooking; nevertheless, once consumed, shogaols are metabolized to paradols in the body [26]. Moreover, the literature on the anti-diabetic activity of paradols and shogaols is scarce. Therefore, it became an important issue to explore the differences of the anti-diabetic potential of these compounds.

AMP-activated protein kinase α (AMPKα) and peroxisome proliferator-activated receptor γ (PPARγ) are two major proteins that regulate cellular glucose and lipid homeostasis, adipocyte differentiation and insulin function in 3T3-L1 adipocytes [27]. Current treatment focuses on targeting both pathways; however, using PPARγ-agonists is often associated with lipid accumulation resulting in obesity [28]. The activation of the PI3K (phosphoinositide 3-kinase)-AKT (protein kinase B) pathway also causes an increase in the glucose uptake by adipocytes [29,30]. Aiming to reveal the glucose-lowering potential of ginger’s active components and their effect on mechanisms related to glucose metabolism, we prepared 10 major components of ginger powder, including 6-, 8-, 10-paradols, 6-, 8-, 10-shogaols, 6-, 8-, 10-gingerols and zingerone. The activity of these compounds in promoting glucose utilization by 3T3-L1 adipocytes or C2C12 myotubes was evaluated. Moreover, the effects on AMPK and related pathways in 3T3-L1 adipocytes were investigated, as well as the glucose-lowering effects in the high-fat diet-fed mice in vivo model.

## 2. Results

### 2.1. Ginger’s Non-Volatile Pungent Components Promote Glucose Utilization in 3T3-L1 Adipocytes

**Figure 1:**
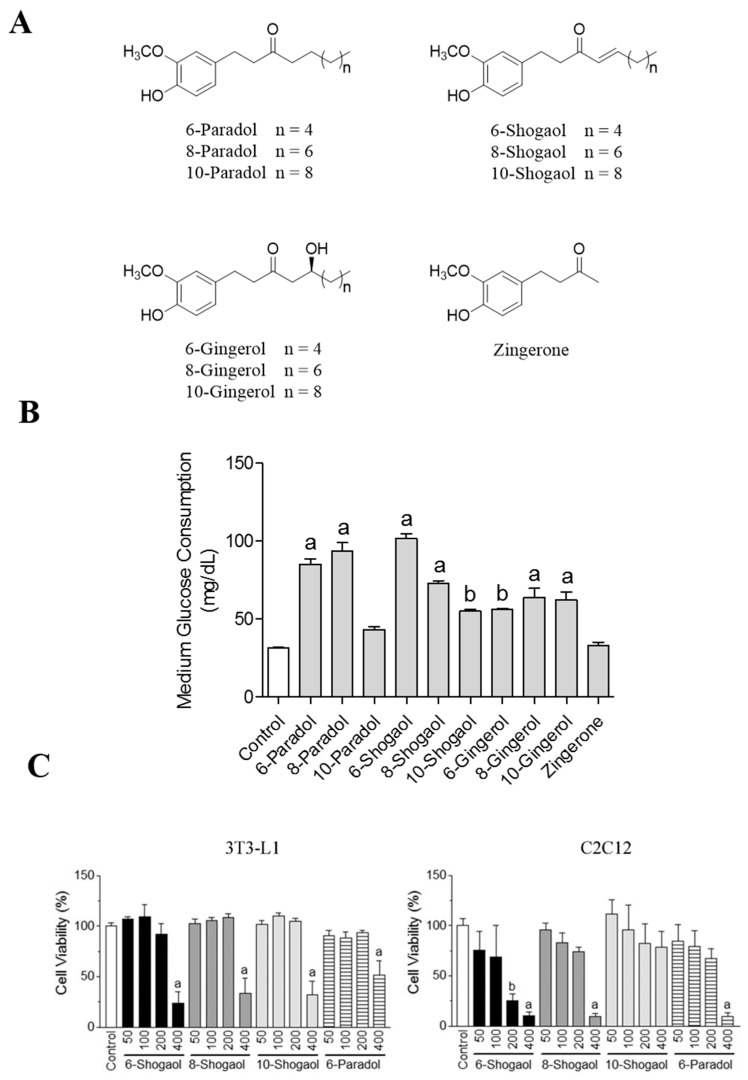
Ginger non-volatile pungent components promote medium glucose consumption by 3T3-L1 adipocytes. (**A**) Chemical structures of ginger’s non-volatile pungent compounds used in this study; (**B**) Medium glucose consumption in 3T3-L1 adipocytes revealing the effect of ginger non-volatile pungent compounds (100 μM). The 3T3-L1 adipocytes were treated with the compounds without insulin in 450 mg/dL d-glucose Dulbecco’s modified Eagle’s medium (DMEM). After 24 h, the remaining glucose concentration of the medium was measured using chemistry analyzer. The data represent the amount of glucose in mg/dL consumed by the cells after 24 h of treatment. Data reflect the mean ± SEM of three independent experiments. ^a^ *p* < 0.001 and ^b^ *p* < 0.01 indicate a significant difference compared with the control group; (**C**) Cell viability of 3T3-L1 (**left**) and C2C12 (**right**) cells after the treatment with 50–400 μM of 6-paradol and 6-, 8-, 10-shogaols for 24 h. The cell viability was detected using water soluble tetrazolium salt (WST-1) reagent (Roche, Basel, Switzerland). Values are the mean ± SEM (*n* = 4). ^a^ *p* < 0.001 and ^b^ *p* < 0.01 compared to the control.

Previous studies indicated that 6-gingerol promoted glucose uptake in 3T3-L1 adipocytes, but the ability of other ginger non-volatile pungent components in promoting cellular glucose utilization is fully understood [19]. In order to evaluate this effect, we used the glucose utilization assay to study the glucose homeostatic activity of 10 gingerol-like compounds (Figure 1A) [31,32]. There was no cytotoxic effect of the 10 gingerol-like compounds on 3T3-L1 adipocytes at the concentration of 100 μM. In the glucose utilization assay, gingerols, shogaols and paradols increased glucose consumption by 3T3-L1 adipocytes (Figure 1B). Each 100 μM sample was tested on the assay for glucose consumption by 3T3-L1 cells. The glucose consumption effect was significantly increased from 31.5 ± 0.3 mg/dL (control) to 33.0 ± 1.8 mg/dL (zingerone), 56.4 ± 0.3 mg/dL (6-gingerol), 64.1 ± 4.7 mg/dL (8-gingerol), 62.4 ± 4.0 mg/dL (10-gingerol), 101.7 ± 2.4 mg/dL (6-shogaol), 73.1 ± 1.1 mg/dL (8-shogaol), 55.0 ± 1.1 mg/dL (10-shogaol), 85.3 ± 2.7 mg/dL (6-paradol), 93.5 ± 4.5 mg/dL (8-paradol) and 43.4 ± 1.5 mg/dL (10-paradol), respectively. Interestingly, zingerone (100 μM) showed no activity in the glucose utilization assay. Among the tested compounds, shogaols and paradols exhibited better activity and therefore were considered for further experimentation. However, insignificant glucose uptake activity of 10-paradol, along with the previously-reported data showing poor bioavailability of 8-paradol [33], motivated us to select 6-paradol and 6-, 8- and 10-shogaols for further investigation. 6-Paradol and 6-, 8- and 10-shogaols were tested for possible cytotoxic effects in concentration range of 50–400 μM showing that these compounds exerted no toxicity on 3T3-L1 adipocytes up to 200 μM and on C2C12 myotubes up to 100 μM (Figure 1C).

### 2.2. 6-Shogaol Promotes Glucose Utilization in 3T3-L1 Adipocytes and C2C12 Myotubes as Well as It Inhibits Lipid Synthesis in 3T3-L1 Adipocytes

**Figure 2:**
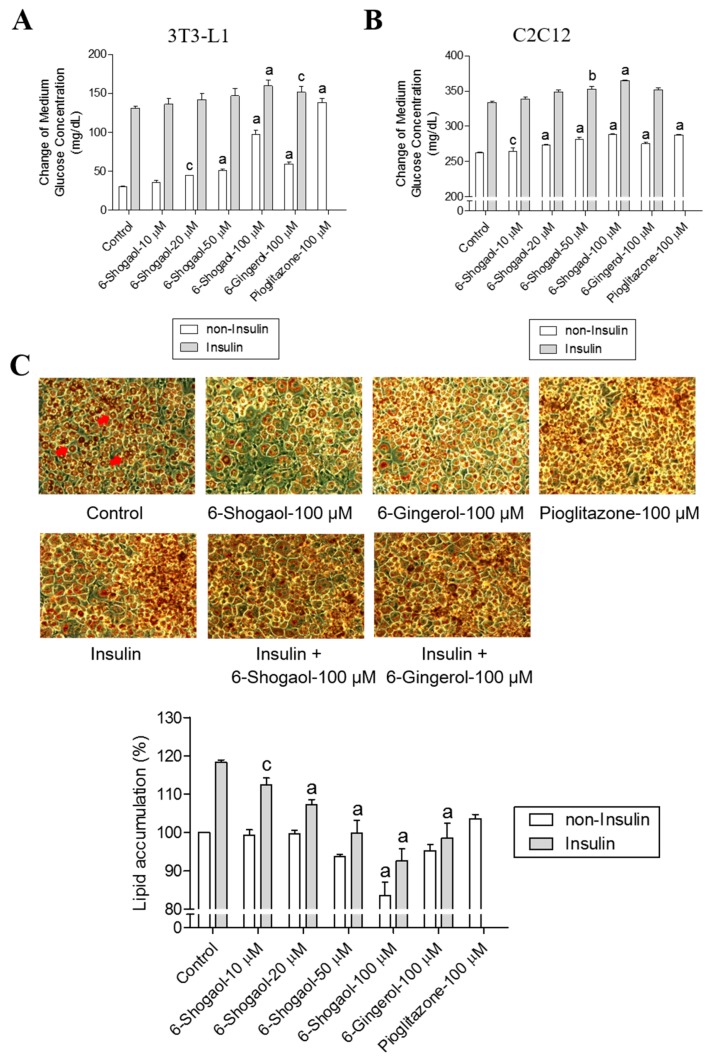
6-Shogaol promotes medium glucose consumption in 3T3-L1 adipocytes and C2C12 myotubes and inhibits lipid synthesis in 3T3-L1 adipocytes. Medium glucose consumption in 3T3-L1 adipocytes (**A**) or C2C12 myotubes (**B**) in response to the treatment of different concentrations of 6-shogaol and 6-gingerol without or with insulin (0.32 μM) in 450 mg/dL d-glucose DMEM. After 24 h, the remaining glucose concentration of the medium was measured using a chemistry analyzer. The data represent the amount of glucose in mg/dL consumed by the cells after 24 h of treatment. 6-Gingerol (100 μM) and pioglitazone (100 μM) were used as the positive controls. (**C**) Oil Red O staining of lipid droplets in 3T3-L1 adipocytes (magnification 200×). Data reflect the mean ± SEM of three independent experiments. The red arrows indicate examples of stained oil drops in adipocytes (see the control picture). ^a^ *p* < 0.001, ^b^ *p* < 0.01 and ^c^ *p* < 0.05 indicate a significant difference compared with the control group.

One of the bioactive compounds of ginger oleoresin is 6-shogaol, which derives its name from “shoga”, the Japanese word for ginger [3]. In the glucose utilization assay, we found that treating 3T3-L1 adipocytes or C2C12 myotubes with 6-shogaol in the absence of insulin increased glucose utilization by the cells in a concentration-dependent manner, with the EC_50_ values of 63.9 ± 3.4 μM and 26.4 ± 1.6 μM, respectively (Figure 2A,B). Moreover, our results revealed that 6-shogaol inhibited lipid synthesis in 3T3-L1 cells, reducing the cellular lipid accumulation in a concentration-dependent manner, with the EC_50_ value of 59.0 ± 2.5 μM (Figure 2C). 6-Gingerol (100 µM) and pioglitazone (100 µM) were used as a positive control in the glucose utilization activity assay (3T3-L1 adipocytes and C2C12 myotubes) and the lipid synthesis inhibition assay (3T3-L1 adipocytes). Pioglitazone increased glucose consumption in 3T3-L1 adipocytes (from 31.5 ± 0.3 mg/dL to 138.5 ± 4.0 mg/dL) and in C2C12 myotubes (from 262.2 ± 1.0 mg/dL to 288.0 ± 0.5 mg/dL), showing better activity in comparison with 6-shogaol (Figure 2A,B). However, pioglitazone did not inhibit lipid synthesis in 3T3-L1 adipocytes (Figure 2C). Treating 3T3-L1 adipocytes or C2C12 myotubes with insulin prior to 6-shogaol resulted in a similar pattern through increasing glucose consumption by the cells, with the EC_50_ values of 41.5 ± 2.2 μM and 21.5 ± 1.3 μM, respectively (Figure 2A,B). 6-Shogaol also inhibited lipid synthesis in 3T3-L1 adipocytes, with the EC_50_ value of 27.1 ± 0.9 μM (Figure 2C). These results encouraged us to compare for the first time the glucose-promoting consumption activity of 6-shogaol with 6-gingerol, which was claimed by previous studies to be the responsible component of ginger’s anti-diabetic activity. We found that 6-shogaol exhibited better activity in promoting glucose utilization and inhibiting lipid synthesis than 6-gingerol, regardless of the presence or absence of insulin (Figure 2).

### 2.3. The Effect of 6-, 8- or 10-Shogaol on Glucose Utilization and Lipid Synthesis

**Figure 3:**
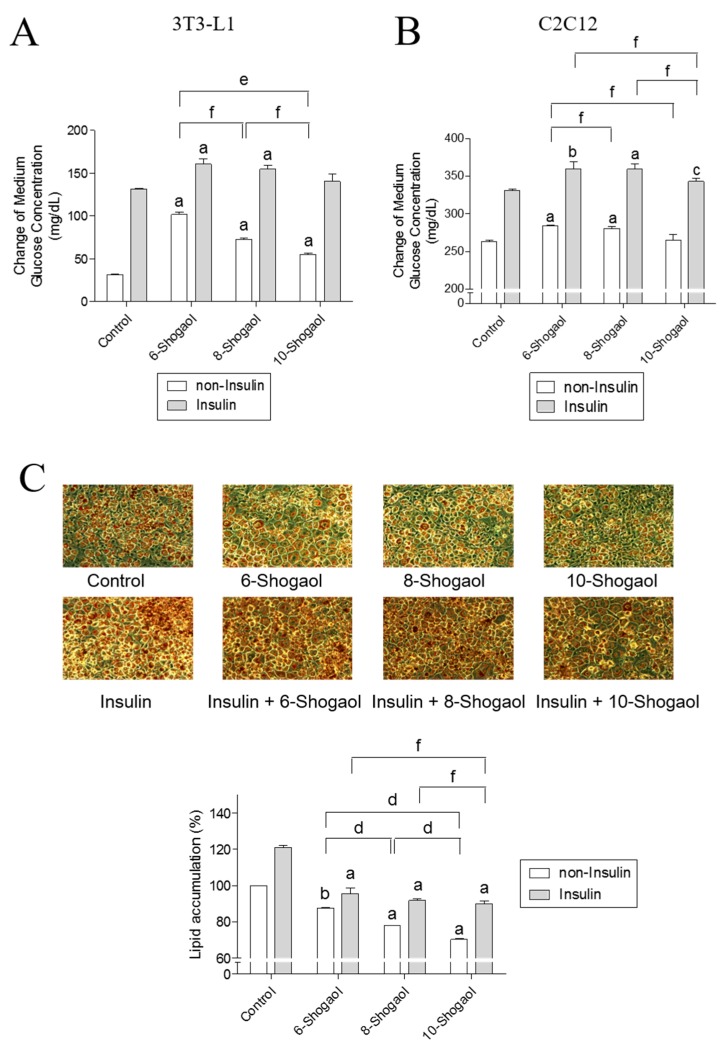
The effect of 6-, 8- or 10-shogaol on glucose utilization and lipid synthesis. Medium glucose consumption in 3T3-L1 adipocytes (**A**) or C2C12 myotubes (**B**) in response to the treatment with 6-, 8- or 10-shogaol. 3T3-L1 adipocytes or C2C12 myotubes were treated with the tested samples with or without insulin treatment (0.32 μM) in 450 mg/dL d-glucose DMEM. After 24 h, the remaining glucose concentration of the medium was analyzed using a chemistry analyzer. The data represent the amount of glucose in mg/dL consumed by the cells after 24 h of treatment. (**C**) Oil Red O staining and measurement of lipid content (magnification 200×). Data reflect the mean ± SEM of three independent experiments. ^a^ *p* < 0.001, ^b^ *p* < 0.01 and ^c^ *p* < 0.05 indicate a significant difference compared with the control group; ^d^ *p* < 0.001, ^e^ *p* < 0.01 and ^f^ *p* < 0.05 indicate a significant difference between the two compared groups.

Previous studies demonstrated that 6-shogaol represents an interesting secondary metabolite targeting different biological pathways in cancer, inflammation, obesity or platelets [34,35,36,37]. However, the glucose uptake activity of 6-shogaol and its longer alkyl chain derivatives, 8- and 10-shogaols, was not studied yet. The effect of the alkyl chain length on the glucose utilization activity of shogaol derivatives was evaluated in 3T3-L1 adipocytes and C2C12 myotubes. When 6-, 8- or 10-shogaol (100 μM) was added to 3T3-L1 adipocytes without insulin, the glucose consumption by the adipocytes increased from 31.5 ± 0.3 mg/dL to 101.7 ± 2.4 mg/dL, 73.1 ± 1.1 mg/dL and 55.0 ± 1.1 mg/dL, respectively (Figure 3A). The treatment of adipocytes with insulin and 6-, 8- or 10-shogaol (100 μM) increased glucose consumption from 131.5 ± 0.9 mg/dL to 160.7 ± 5.2 mg/dL, 154.8 ± 3.9 mg/dL and 140.5 ± 7.3 mg/dL, respectively (Figure 3A). Similarly, the addition of 6-, 8- or 10-shogaol (100 μM) to C2C12 myotubes without insulin resulted in an increase of myotubes glucose consumption from 263.2 ± 1.1 mg/dL to 284.2 ± 0.7 mg/dL, 280.7 ± 2.1 mg/dL and 265.0 ± 6.3 mg/dL, respectively (Figure 3B). The effect of shogaol derivatives (6-, 8- or 10-shogaol, 100 μM) on glucose consumption in 3T3-L1 adipocytes treated with insulin was also evaluated showing an increase in the consumption from 331.2 ± 1.1 mg/dL to 360.0 ± 7.2 mg/dL, 359.5 ± 5.4 mg/dL and 343.1 ± 3.5 mg/dL, respectively (Figure 3B). Moreover, the addition of 6-, 8- or 10-shogaol (100 μM) to 3T3-L1 adipocytes reduced lipid accumulation. In insulin-free groups, the lipid accumulation decreased from 100% (the control group) to 87.6% ± 0.1%, 77.9% ± 0.2% and 70.2% ± 0.3%, respectively. In the insulin-treated groups, the lipid accumulation decreased from 120% ± 3.1% (the control group) to 95.5% ± 2.6%, 91.8% ± 0.8% and 90.0% ± 1.3%, respectively (Figure 3C).

### 2.4. 6-Paradol Promotes Glucose Utilization in 3T3-L1 Adipocytes and C2C12 Myotubes as Well as It Inhibits Lipid Synthesis in 3T3-L1 Adipocytes

**Figure 4:**
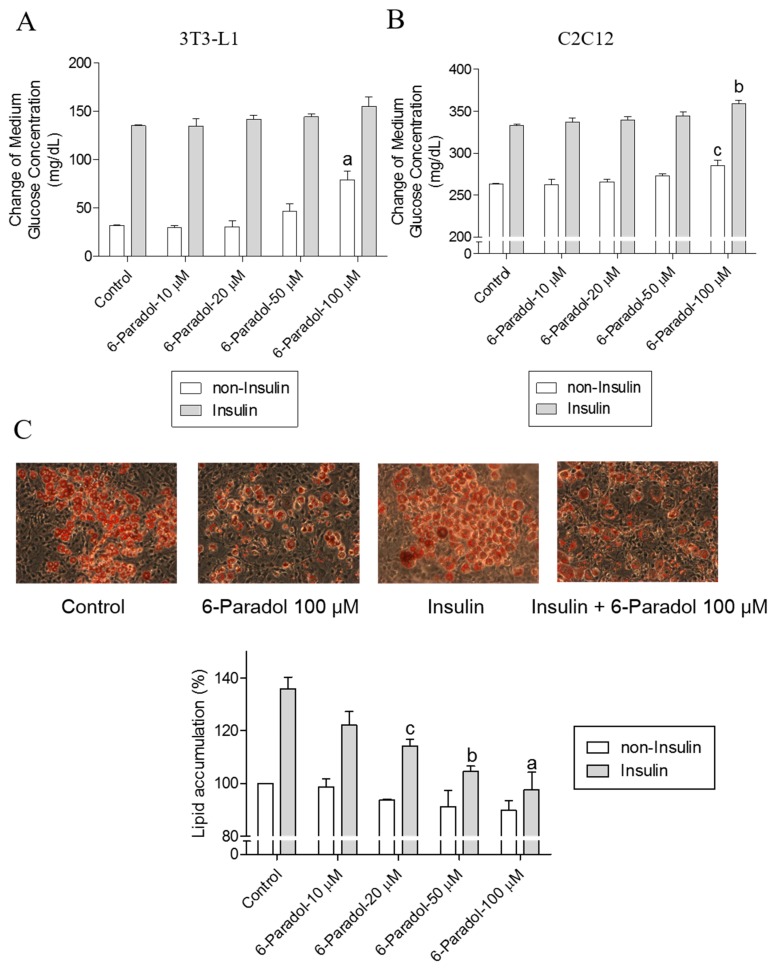
The effect of 6-paradol on glucose utilization and lipid synthesis. Medium glucose consumption in 3T3-L1 adipocytes (**A**) or C2C12 myotubes (**B**) in response to the treatment with 6-paradol. 3T3-L1 adipocytes or C2C12 myotubes were treated with the tested samples with or without insulin (0.32 μM) in 450 mg/dL d-glucose DMEM. After 24 h, the remaining glucose concentration of the medium was measured using a chemistry analyzer. The data represent the amount of glucose in mg/dL consumed by the cells after 24 h of treatment. (**C**) Oil Red O staining and measurement of lipid content (magnification 200×). Results are the mean ± SEM of three independent experiments. ^a^ *p* < 0.001, ^b^ *p* < 0.01 and ^c^ *p* < 0.05 indicate a significant difference compared with the control group.

Although 6-paradol is only a minor constituent of ginger, mainly formed from 6-gingerol via 6-shogaol, 6-shogaol was reported to be almost completely metabolized into 6-paradol in mice [26]. Therefore, we evaluated the concentration-dependent effects of 6-paradol on glucose utilization. In 3T3-L1 adipocytes, 6-paradol increased glucose utilization by the cells in a concentration-dependent manner, with insulin EC_50_ 53.2 ± 5.5 μM and without insulin EC_50_ 65.4 ± 5.3 μM. The similar effect was also observed in C2C12 myotubes, with insulin EC_50_ 54.2 ± 4.7 μM and without insulin EC_50_ 54.9 ± 3.3 μM (Figure 4A,B). Moreover, our results revealed that 6-paradol inhibited lipid synthesis in 3T3-L1 cells, reducing the cellular lipid accumulation in a concentration-dependent manner, with insulin EC_50_ 13.3 ± 3.3 μM and without insulin EC_50_ 29.8 ± 1.4 μM (Figure 4C).

### 2.5. The Mechanisms of 6-Shogaol and 6-Paradol in Regulating Glucose Utilization and Lipid Accumulation in 3T3-L1 Adipocytes

**Figure 5:**
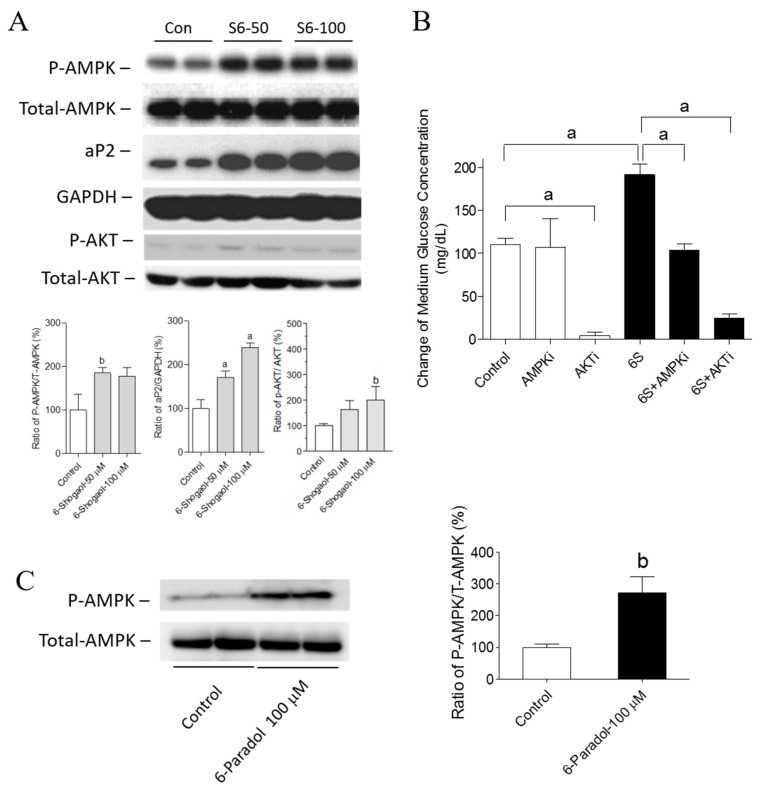
The effects of 6-shogaol on AKT and AMPK phosphorylation and aP2 protein expression and of 6-paradol on AMPK phosphorylation. (**A**) Differentiated 3T3-L1 adipocytes were treated with 6-shogaol or vehicle (control) for 48 h. Protein levels of P-AMPK, total-AMPK, P-AKT, total-AKT, aP2 and GAPDH were determined by Western blot analysis and expressed as a percentage of the control. (**B**) The effect of 6-shogaol on glucose consumption co-treated with specific kinase inhibitors of AMPK or AKT in 3T3-L1 cells. After 24 h, the remaining glucose concentration of the medium was measured using a chemistry analyzer. The data represent the amount of glucose in mg/dL consumed by the cells after 24 h of treatment. (**C**) Differentiated 3T3-L1 adipocytes were treated with 6-paradol or vehicle (control) for 48 h. Protein levels of P-AMPK and total-AMPK were determined by Western blot analysis and expressed as a percentage of the control. Results are the mean ± SEM of three independent experiments. ^a^ *p* < 0.001 and ^b^ *p* < 0.01 indicate a significant difference compared with the control group.

In order to investigate the molecular pathways in glucose metabolism regulated by the treatment of 6-shogaol and 6-paradol, the major molecular targets in hyperglycemia were investigated. First, the protein level of phosphorylated AMPKα, which represents the enzymatic activity of AMPKα, was analyzed and compared with the protein level of total AMPKα. We found that AMPKα phosphorylation was induced by 6-shogaol (Figure 5A). We also investigated the effect of 6-shogaol on AKT activation, and our results showed that AKT phosphorylation was increased (Figure 5A). In addition, we used specific kinase inhibitors of AMPK and AKT to prove the involvement of AMPK and AKT pathways in the regulatory effect of 6-shogaol on glucose consumption (Figure 5B). The increased glucose consumption by 6-shogaol was reverted by an AMPK inhibitor. This indicated that the glucose consumption effect of 6-shogaol was dependent on the AMPK pathway. The AKT inhibitor suppressed both basal and 6-shogaol-enhanced glucose consumption, indicating that the AKT pathway is essential for the glucose uptake in both basal and treatment conditions. In addition, we investigated whether AMPKα is activated by 6-paradol, the major metabolite of 6-shogaol, and we found that AMPKα phosphorylation was induced by 6-paradol (Figure 5C, see Figure S1 for 10–100 μM). Furthermore, we evaluated the effect of 6-shogaol on the protein expressions of adipocyte fatty acid binding protein 4 (aP2), a down-stream protein of PPARγ. We observed that aP2 protein expression was increased following the treatment by 6-shogaol (Figure 5A). The effects on the expression of other proteins, including sterol regulatory element binding protein-1 (SREBP-1), CCAAT enhancer binding protein (C/EBP-α), fatty acid synthase (FAS) and glucose transporter type 4 (GLUT-4) protein, can be found in the Supporting Information (Figure S2).

### 2.6. The Anti-Hyperglycemic Effect of 6-Paradol in the High-Fat Diet Mouse Model

**Figure 6:**
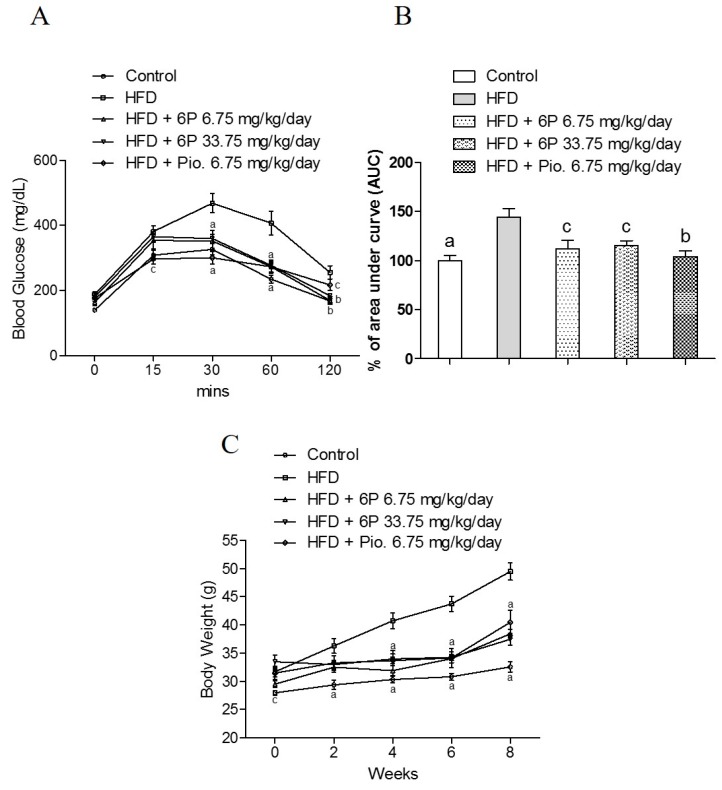
6-Paradol alleviates blood glucose and body weight in high-fat diet-fed mice. (**A**) Effect of 6-paradol on glucose tolerance in mice fasted for 2 h as determined by the oral glucose tolerance test (OGTT); (**B**) area under the curve (AUC) for the OGTT test; (**C**) body weight within eight weeks. Data reflect the mean ± SEM of three independent experiments. ^a^ *p* < 0.001, ^b^ *p* < 0.01 and ^c^ *p* < 0.05 indicate a significant difference compared with the HFD group. Control represents the group of normal- diet-fed mice (*n* = 9); HFD represents the group of high-fat diet-fed mice (*n* = 8); HFD + 6P 6.75 mg/kg/day represents the group of high-fat diet-fed mice fed with the 6-paradol (6.75 mg/kg/day) treatment (*n* = 7); HFD + 6P 33.75 mg/kg/day represents the group of high-fat diet-fed mice fed with the 6-paradol (33.75 mg/kg/day) treatment (*n* = 6); HFD + Pio. 6.75 mg/kg/day represents the group of high-fat diet-fed mice fed with the pioglitazone (6.75 mg/kg/day) treatment (*n* = 6).

After the evaluation of the glucose-lowering potential using the in vitro model, the anti-hyperglycemic effect of 6-paradol, representing a major metabolite of 6-shogaol [26], was further studied using the high-fat diet mouse in vivo model. We used the oral glucose tolerance test (OGTT) to examine the effect of 6-paradol on hyperglycemia, which was developed in non-hyperglycemic mice by high-fat diet (HFD). The high-fat diet (HFD) group had significantly higher blood glucose as compared with the normal diet group (control) (Figure 6A). Compared with the HFD group, the treatment with 6-paradol low dose (6.75 mg/kg/day) or high dose (33.75 mg/kg/day), as well as with pioglitazone (6.75 mg/kg/day) significantly lowered the blood glucose at the 30-, 60- and 120-min time points. The effect of 6-paradol on high-fat diet-fed mice was clearly illustrated in the area under the curve (AUC) showing that the treatment with either a low or high dose of 6-paradol significantly decreased blood glucose to a similar level of the positive control, pioglitazone (Figure 6B). The body weight was significantly lowered by the treatment for eight weeks with 6-paradol either at a low dose or a high dose, and the treatment for four weeks was already effective (Figure 6C). Table 1 summarizes the biochemical data of the mice. Fasting glucose and the total cholesterol levels of the normal diet group (control) were significantly different from the high-fat diet (HFD) group. In comparison with the HFD group, the fasting glucose of the 6-paradol-treated (high dose) group was significantly lower, supporting the anti-hyperglycemic activity of 6-paradol demonstrated by the in vitro model. The elevated total cholesterol levels in high fat diet-fed mice were significantly decreased by 6-paradol treatment in both the low and high dose groups. Moreover, 6-paradol (high dose) partially reversed the elevation of triglycerides in high fat-fed diet mice. Plasma ALT level was increased in the HFD group; in contrast, 6-paradol (both high and low dose) significantly prevented the elevation of plasma ALT levels, which indicated that 6-paradol had no liver toxicity throughout the eight-week administration regimen (Table 1). On the other hand, creatinine levels were slightly increased in the high dose 6-paradol-treated group.

## 3. Discussion

Recent studies indicated that certain ginger oleoresin pungent components are unstable and can be easily converted from one compound to another [5]. Among these compounds is 6-gingerol, which is highly concentrated in the fresh rhizomes and is considered thermally labile due to the presence of the β-hydroxyl-keto group. A significant amount of 6-gingerol is converted to 6-shogaol through dehydration and to a lesser extent to zingerone through a retro aldol reaction. Moreover, the hydrogenation of 6-shogaol can lead to the formation of 6-paradol. These changes usually occur upon drying, prolonged storage or cooking [5,25]. The dynamic conversion of ginger’s active components and the differences in their activity in promoting glucose utilization could account for the discrepancy in the anti-hyperglycemic activity results of ginger’s non-standardized extracts. Our results also indicated that the length of the alkyl chain plays an important role in the anti-hyperglycemic activity. Zingerone possesses the basic phenolic structure of gingerols, shogaols and paradols, but lacks their unbranched alkyl chains. Our data showed that zingerone demonstrated an insignificant effect compared to those of other derivatives. The importance of the alkyl chain length on bioactivity was also reported in previous studies focusing on the antioxidant activity of ginger secondary metabolites and the anti-obesity activity of paradol analogues [33,38].

Previous reports indicated that gingerols possess an insulin-independent glucose uptake effect in L6 muscle cells and an insulin-dependent effect in 3T3-L1 adipocytes [19,20]. Our results showed that both 6-shogaol and 6-paradol improved glucose utilization in both adipocytes and muscle cells with or without insulin. This effect indicates that 6-shogaol and 6-paradol enhance glucose utilization via a distinct pathway independent of insulin. Furthermore, the results demonstrated the inhibitory effect of 6-shogaol and 6-paradol on lipid synthesis in 3T3-L1 adipocytes. The close relationship between diabetes and obesity implies that an ideal anti-diabetic agent should act in a dual mode through targeting high blood glucose, as well as lipid accumulation [39]. Pioglitazone, an anti-diabetic agent, acts by decreasing blood glucose, but it does not inhibit lipid accumulation in adipocytes [40]. However, our preliminary results revealed that 6-shogaol and 6-paradol not only promoted glucose utilization, but they also inhibited lipid synthesis by adipocytes.

6-, 8- or 10-shogaol possess the same aryl moiety, but differ in the length of their unbranched alkyl chain. Accumulated evidence suggested that the side chain length of shogaol derivatives influences their biological activity [41]. In our study, we observed that a shorter carbon side chain (6-shogaol and 8-shogaol) exerted stronger activity in promoting glucose utilization. Interestingly, stronger inhibition of lipid synthesis was observed by the treatment with longer carbon side chain compounds (10-shogaol and 8-shogaol). The aforementioned findings suggested that these compounds have multiple targets in 3T3-L1 cells and thus could affect different functional pathways.

AMPK is an important kinase in homeostasis acting as a metabolic master regulating glucose and lipid metabolism [42]. The activation of AMPK by pharmacological agents holds a great potential for reversing metabolic abnormalities associated with type 2 diabetes, for instance hyperglycemia [43]. According to our results, the effect of 6-paradol and 6-shogaol on glucose uptake was associated with the activation of the AMPK pathway. PPARγ regulates the transcription of many genes, for instance fatty acid-binding protein 4 (adipocyte protein 2; aP2), which is involved in glucose and lipid metabolism [44,45]. Previously, Yasuka et al. demonstrated that 6-shogaol is a potent PPARγ agonist, in contrast to 6-gingerol, which did not activate PPARγ [35]. In agreement with Yasuka group showing increased aP2 gene expression, we found that aP2 protein expression was increased upon treating 3T3-L1 adipocytes with 6-shogaol. It is well known that the PPARγ ligand will increase glucose consumption, but with the side-effect of increased lipid accumulation [28]. According to our results this side effect may be reversed by the 6-shogaol-activated AMPK pathway, resulting in the inhibition of lipid accumulation. Moreover, the effect on both pathways may contribute to the increase of the glucose consumption. Nevertheless, to understand whether experimental conditions and stress may have influenced the observed effects of 6-paradol and shogaols on glucose utilization and AMPK activation, further studies should be conducted.

The variability in previously-reported results on ginger biological activity may be attributed to a dynamic change in its active components upon storing or cooking [46]. In vitro model of pH- and temperature-dependent transition of 6-gingerol to 6-shogaol and vice versa showed that the reaction is favored towards gingerol under conditions similar to those in the stomach (37 °C, acidic pH, equilibrium at 96 h) [46]. Although the anti-hyperglycemic activity of gingerol and shogaol was similar according to our results, zingerone, which is formed from gingerol upon drying, showed insignificant effects. Previous studies reported that shogaols are extensively metabolized in in vivo models. Interestingly, paradol is formed upon feeding with shogaol as reported in a study on metabolism of 6-shogaol in mice [26]. In another report, it was clearly evidenced that 6-paradol was effectively absorbed into the enterocyte following oral ingestion. On the other hand, 8- or 12-paradol had only poor bioavailability [33]. The anti-obesity activities of 6-, 8- and 12-paradol decreased with a longer alkyl chain length. The study showed that 6-paradol significantly decreased body weight and lipogenesis, via activation of thermogenesis in brown adipose tissue (BAT) [33]. Therefore, 6-paradol was chosen for the in vivo experimentation. According to our results, 6-paradol exhibited a significant glucose-lowering effect (OGGT data, Figure 6) and decreased body weight. Taken together, our study indicated that 6-paradol possesses good anti-hyperglycemic activity, therefore it may serve as a novel target for the development of anti-obesity and anti-hyperglycemic functional food.

## 4. Materials and Methods

### 4.1. Chemicals

Zingerone (88787, >98%), 6-gingerol (G1046, >98%), 8-gingerol (01514, >95%), 10-gingerol (G5798, >95%), d-glucose, 3-isobutyl-1-methylxanthine, dexamethasone, insulin, ethanol (EtOH), Oil Red O, propylene glycol, dimethyl sulfoxide (DMSO), pioglitazone, aldehydes (starting materials for shogaols synthesis), AKT inhibitor, AMPK inhibitor compound C and all other chemicals were of analytical grade and were obtained from Sigma-Aldrich Inc. (Saint Louis, MO, USA). Low glucose Dulbecco’s Modified Eagle Medium (DMEM, low glucose; Catalog No.: 12320), penicillin/streptomycin and fetal bovine serum (FBS), horse serum, trypsin-EDTA were bought from Invitrogen (Carlsbad, CA, USA). Anti-AMPK, anti-phospho-AMPK, anti-aP2, anti-C/EBP-α, anti-FAS, anti-GLUT-4, anti-AKT, anti-phospho-AKT and anti-GAPDH antibodies were purchased from Cell Signaling Technology (Danvers, MA, USA). Anti-SREBP-1 was purchased from Santa Cruz Biotechnology, Inc. (Dallas, TX, USA).

### 4.2. Preparation of 6-Shogaol, 8-Shogaol, 10-Shogaol, 6-Paradol, 8-Paradol and 10-Paradol

Dry tetrahydrofuran (THF) was added to a flask with zingerone (1.0 equiv.) under inert atmosphere. The reaction was cooled to −78 °C; potassium tertiary butoxide (*t*BuOK, 2.5 equiv.) was added in a single portion, and the mixture was kept stirring for 1 h. For the preparation of each target compound, the corresponding aldehyde starting material (1.2 equiv.) was added slowly to the reaction mixture. The reaction was kept stirring for another 2 h and finally quenched by water. After extracting the mixture with dichloromethane, the organic part was evaporated under vacuum. The residue was purified by column chromatography using *n*-hexane/ethyl acetate (4:1) to afford the corresponding racemic gingerol. The synthesized gingerol racemate (1.0 equiv.) was dissolved in dry benzene followed by the addition of *p*-toluenesulfonic acid (0.4 equiv.). The mixture was refluxed for 1 h, then quenched by water. After extracting the mixture with dichloromethane, the organic layer was dried under vacuum. The residue was purified by column chromatography using *n*-hexane/ethyl acetate (4:1) as the solvent system to afford the corresponding shogaol.

For the synthesis of the three paradol derivatives, 6-, 8- or 10-shogaol (100.0 mg) was dissolved in 5 mL ethyl acetate. To this solution, 10% palladium on carbon (Pd/C, 82.0 mg) was added, and the mixture was degassed with hydrogen. The reaction was stirred under hydrogen atmosphere (1 atm) at room temperature for 12 h. After the reaction completion, the solution was filtered through a short pad of silica gel and washed with dichloromethane. The organic solvent was evaporated under vacuum, and the residue was purified by column chromatography eluted with *n*-hexane/ethyl acetate (4:1) to afford the corresponding paradol. The yield, purity, nuclear magnetic resonance data, ^1^H NMR and ^1^C NMR data for 6-shogaol, 8-shogaol, 10-shogaol, 6-paradol, 8-paradol and 10-paradol can be found in the Supplementary Materials.

### 4.3. Cell Culture and Differentiation

Mouse 3T3-L1 pre-adipocytes (BCRC#60159) and C2C12 muscle myoblast cells (BCRC#60083) were purchased from the Bioresource Collection and Research Center (BCRC, Food Industry Research and Development Institute, Hsinchu City, Taiwan). 3T3-L1 and C2C12 cells were seeded on a 10-cm culture dish (Corning Incorporated, Corning, NY, USA) and maintained in low glucose Dulbecco’s Modified Eagle’s Medium (DMEM) with 10% (*v*/*v*) fetal bovine serum (FBS) and 1% penicillin-streptomycin (P/S) in a humidified atmosphere of 95% air and 5% CO_2_ at 37 °C. For each experiment, the cells were transferred to a 6-cm culture dish (Corning Incorporated, Corning, NY, USA) and cultured under the same condition. After reaching 100% confluence, the 3T3-L1 cells were incubated for an additional 48 h. For the differentiation step, 3T3-L1 pre-adipocyte cells were then cultured using the differentiation medium (low glucose DMEM containing 450 mg/dL d-glucose, 10% FBS, 0.32 μM insulin, 0.5 mM 3-isobutyl-1-methylxanthine and 0.5 μM dexamethasone) for 48 h [31]. When C2C12 muscle myoblast cells reached 100% confluence, the cells were incubated with the differentiation medium (low glucose DMEM containing 450 mg/dL d-glucose and 2% horse serum). The differentiation medium was replaced over two-day regular intervals until 90% of the cells were differentiated to form myotubes (approximately 7 days) [47]. The cytotoxicity of 6-paradol and shogaols on 3T3-L1 cells was determined using the water soluble tetrazolium salt (WST-1) assay [48].

### 4.4. Medium Glucose Utilization Assay

Following the differentiation step, 3T3-L1 or C2C12 cells were cultivated in high glucose medium (low glucose DMEM containing 450 mg/dL d-glucose, 10% FBS and 1% P/S) and treated with or without the tested compounds. After 24 h, the medium glucose utilization activity was determined by measuring the medium glucose concentration using a Roche Cobas Integra 400 Chemistry Analyzer (Roche Diagnostics, Taipei, Taiwan) [31,32]. The analyzer coefficient of variation (CV) was 0.62%–0.92% within-run and 1.1%–1.2% between days. The differentiated cells without the tested compounds were assigned as the negative control. Pioglitazone were used as the positive controls. The insulin powder was dissolved in 0.01 M acetic acid (pH 3.0) to provide a 0.01 M stock solution and then diluted with distilled water. Compounds were dissolved in DMSO to prepare 100 mM stock solutions and then diluted in DMSO; the final concentration of DMSO in the medium was 0.1%.

### 4.5. Oil Red O Staining

After the cells were differentiated for two days, 3T3-L1 adipocytes were cultured with high glucose medium and treated with the tested compounds for another six days. The medium with the tested compounds was changed on two-day regular intervals. After six days, the cells were washed twice with phosphate buffered saline (PBS) and fixed with 4% paraformaldehyde for 30 min at room temperature and finally washed with PBS. The fixed cells were stained with 0.5% Oil Red O (Sigma-Aldrich Inc.) in propylene glycol for one hour and then washed with PBS. The stained lipid droplets were photographed by light microscopy. The accumulated lipid was extracted in 2 mL of pure isopropanol and the accumulated amount was measured by reading the absorbance at OD 490 nm.

### 4.6. Western Blot Analysis

After 3T3-L1 adipocytes were differentiated for two days as described above, the cells were cultured with high glucose medium. On Days 4 and 5, the cells were cultured in high glucose medium with or without 50–100 μM 6-, 8- or 10-shogaol and 6-paradol. On Day 6, the cells were harvested for total protein extraction. Total protein samples were prepared using the M-PER Mammalian Protein Extraction Reagent (Thermo Scientific, Rockford, IL, USA) supplemented with protease inhibitors (Roche Diagnostics, Taipei, Taiwan). The protein concentration was measured using the Protein Assay Kit (Bio-Rad Laboratories Inc., Hercules, CA, USA). Thirty micrograms of protein samples were loaded on 10% or 15% SDS-PAGE, transferred onto a polyvinylidene difluoride (PVDF) membrane (Merck Millipore, Billerica, MA, USA) and blocked with 5% nonfat milk in Tween-Tris-buffered saline (TBST) solution for 30 min. The membrane was incubated with anti-AMPK, anti-phospho-AMPK, anti-aP2, anti-SREBP-1, anti-C/EBP-α, anti-FAS, anti-GLUT-4, anti-AKT, anti-phospho-AKT or anti-GAPDH primary antibody overnight, followed by the incubation with the secondary antibody for 2 h. The blots were visualized using the Western Breeze Chemiluminescent Western Blot Immunodetection Kit (PerkinElmer, Norwalk, CT, USA) and quantified using the ImageJ software (National Institutes of Health, Bethesda, MD, USA).

### 4.7. Animal Model

A type 2 diabetic model was utilized according to Srinivasan and Ramarao [49]. Simply, non-obese and non-diabetic C57BL/6J mice were fed a high-fat diet to induce hyperglycemia. Eight-week-old male C57BL/6J mice were obtained from BioLASCO Technology (Charles River Technology, BioLASCO Taiwan Co., Ltd., Taipei, Taiwan). All mice received standard animal care under the supervision of the Institutional Animal Care and Use Committee of Kaohsiung Medical University, Taiwan. Mice were caged in an air-conditioned animal facility at 23 °C on a 12-h light/dark cycle and were maintained with free access to water and food. Animals were fed either a normal chow diet consisting of 11% fat, 65% carbohydrate and 24% protein expressed as a percentage of total kcal (Maintenance diet 1320, Altromin Spezialfutter GmbH & Co. KG, Lage, Germany) or a high-fat diet consisting of 45% fat, 35% carbohydrate and 20% protein (D12451, Research Diets, Inc., New Brunswick, NJ, USA). After 4 weeks on either diet, mice were divided into 5 groups: (i) control (*n* = 9); (ii) high-fat diet mice (*n* = 9); (iii) high-fat diet mice plus 6-paradol treatment (6.75 mg/kg/day; *n* = 7); (iv) high-fat diet mice plus 6-paradol treatment (33.75 mg/kg/day; *n* = 6); and (v) high-fat diet mice plus pioglitazone treatment (6.75 mg/kg/day; *n* = 6). The doses chosen for the experiment were based on the clinically-relevant dose of pioglitazone for the human converted to mice model by a coefficient of 9.01 (pioglitazone, 6.75 mg/kg/day and 6-paradol, 6.75 mg/kg/day and 5-fold, 33.75 mg/kg/day) [50]. 6-Paradol and pioglitazone were dissolved in the vehicle, which contained 0.3 mL *N*,*N*-dimethylacetamide, 0.9 mL polyethylene glycol (PEG)-400, 1.2 mL tetraglycol and 0.6 mL water, due to their water-insoluble properties [51]. Mice were treated orally via gastric gavage for 8 weeks, three times per week (Monday, Wednesday and Friday). The control group was gavaged with the vehicle. Throughout the experiment, every second week, the body weight and blood glucose of mice were measured. Blood glucose from the tail tip (before meal) was detected by the ACCU-CHEK blood glucose meter (Roche Diagnostics, Taipei, Taiwan). Animals were sacrificed after 8 weeks of treatment, two days after the last drug administration. After fasting overnight, mice were euthanized by intraperitoneal injection with Zoletil (10 mg/kg), the anesthetic drug (Virbac, Carros, France). Blood samples were collected from the hearts at the time of sacrifice and centrifuged at 3000 rpm for 15 min. Plasma (supernatant) was stored in a −20 °C freezer. The biochemistry data including fasting plasma glucose, triglyceride, total cholesterol, alanine aminotransferase and creatinine, were obtained using the Roche Cobas Integra 400 Chemistry Analyzer (Roche Diagnostics, Taipei, Taiwan).

### 4.8. Oral Glucose Tolerance Test

To observe whether the compounds could improve glucose intolerance after 8 weeks of treatment, we performed the oral glucose tolerance test (OGTT). Mice were starved for 5–7 h, following the administration of 2.0 g/kg glucose via gastric gavage (0 min). Blood samples were taken from the tail tips at 0, 15, 30, 60 and 120 min. Blood glucose concentration was measured using an ACCU-CHEK blood glucose meter, and the area under curve (AUC) of OGTT was analyzed by GraphPad Prism 5 software (GraphPad Software, Inc., La Jolla, CA, USA).

### 4.9. Statistical Analysis

A minimum of three independent experiments was performed per procedure. Data were analyzed by one-way analysis of variance followed by the Bonferroni test using GraphPad Prism 5 software (GraphPad Software, Inc.). All data are presented as the mean ± standard error of the mean (SEM). Values with ^a^ *p* < 0.001, ^b^ *p* < 0.01 and ^c^ *p* < 0.05 were regarded as statistically significant.

## 5. Conclusions

In conclusion, the glucose utilization-promoting activities of gingerols, shogaols, paradols and zingerone were compared for the first time in the current study. 6-Shogaol and 6-paradol showed potent activity in stimulating glucose utilization by 3T3-L1 adipocytes and C2C12 myotubes. The effect was attributed to an increase in AMPK phosphorylation. Furthermore, 6-paradol, a metabolite of 6-shogaol, was utilized in the high-fat diet mouse model. 6-Paradol decreased blood glucose, cholesterol and body weight. Our findings suggested that previously-reported different effects of ginger powder on hyperglycemia may be attributed to the degradation and metabolic changes of ginger compounds. The results also indicated that 6-paradol could be considered as an active anti-hyperglycemic component of ginger.

## Figures and Tables

**Table 1:** Biochemical data of the mice.

Parameters	Control	HFD	HFD + 6-Paradol (6.75 mg/kg/day)	HFD + 6-Paradol (33.75 mg/kg/day)	HFD + Pioglitazone (6.75 mg/kg/day)
	*n* = 9	*n* = 8	*n* = 7	*n* = 6	*n* = 6
Fasting glucose (mg/dL)	176.2 ± 7.5 ^c^	242.1 ± 13.5	201.93 ± 17.8	151 ± 12.3 ^b^	210 ± 27.8
Triglycerol (mg/dL)	49.2 ± 4.2	59.4 ± 4.9	56.3 ± 4.4	51.4 ± 8.0	64.3 ± 8.3
T-CHO (mg/dL)	65.9 ± 3.1 ^a^	148.1 ± 8.6	111 ± 3.4 ^a^	104.4 ± 7.8 ^a^	119.3 ± 5.2 ^b^
ALT (U/L)	36.5 ± 2.7 ^b^	103.1 ± 23.3	25.2 ± 4.8 ^a^	38.6 ± 8.2 ^b^	42.45 ± 8.8 ^c^
Creatinine (mg/dL)	0.17 ± 0.02	0.18 ± 0.01	0.20 ± 0.01	0.27 ± 0.03 ^b^	0.21 ± 0.02

Values are the mean ± SD. Data were analyzed by one-way analysis of variance followed by the Bonferroni test. ^a^ *p* < 0.001 compared to HFD; ^b^ *p* < 0.01 compared to HFD; ^c^ *p* < 0.05 compared to HFD. HFD, high-fat-diet, T-CHO, total cholesterol, ALT, alanine aminotransferase.

## Supplementary Materials

Supplementary materials can be found at www.mdpi.com/1422-0067/18/1/168/s1.

## Abbreviations

HFD	high-fat diet
GLUT4	glucose transporter type 4
LDL	low-density lipoprotein
VLDL	very-low-density lipoprotein
DMSO	dimethyl sulfoxide
EtOH	ethanol
DMEM	Dulbecco’s Modified Eagle Medium
FBS	fetal bovine serum
THF	tetrahydrofuran
*t*BuOK	potassium tertiary butoxide
Pd/C	palladium on carbon
AMPK	5′ adenosine monophosphate-activated protein kinase
PPAR	peroxisome proliferator-activated receptor
aP2	adipocyte fatty acid binding protein 4
SREBP-1	sterol regulatory element binding protein-1
C/EBP-α	CCAAT enhancer binding protein
FAS	fatty acid synthase
OGTT	oral glucose tolerance test
BAT	brown adipose tissue

## References

[1] Butt M.S., Sultan M.T. (2011). Ginger and its health claims: Molecular aspects. Crit. Rev. Food Sci. Nutr..

[2] Shukla Y., Singh M. (2007). Cancer preventive properties of ginger: A brief review. Food Chem. Toxicol..

[3] Al-Suhaimi E.A., Al-Riziza N.A., Al-Essa R.A. (2011). Physiological and therapeutical roles of ginger and turmeric on endocrine functions. Am. J. Chin. Med..

[4] White B. (2007). Ginger: An overview. Am. Fam. Physician.

[5] Jiang H., Xie Z., Koo H.J., McLaughlin S.P., Timmermann B.N., Gang D.R. (2006). Metabolic profiling and phylogenetic analysis of medicinal *Zingiber* species: Tools for authentication of ginger (*Zingiber officinale* Rosc). Phytochemistry.

[6] Ali B.H., Blunden G., Tanira M.O., Nemmar A. (2008). Some phytochemical, pharmacological and toxicological properties of ginger (*Zingiber officinale* Roscoe): A review of recent research. Food Chem. Toxicol..

[7] Nicoll R., Henein M.Y. (2009). Ginger (*Zingiber officinale* Roscoe): A hot remedy for cardiovascular disease?. Int. J. Cardiol..

[8] Altman R.D., Marcussen K.C. (2001). Effects of a ginger extract on knee pain in patients with osteoarthritis. Arthritis Rheum..

[9] Chrubasik S., Pittler M.H., Roufogalis B.D. (2005). Zingiberis rhizoma: A comprehensive review on the ginger effect and efficacy profiles. Phytomedicine.

[10] Chen B.H., Wu P.Y., Chen K.M., Fu T.F., Wang H.M., Chen C.Y. (2009). Antiallergic potential on RBL-2H3 cells of some phenolic constituents of *Zingiber officinale* (ginger). J. Nat. Prod..

[11] Imam K. (2012). Clinical features, diagnostic criteria and pathogenesis of diabetes mellitus. Adv. Exp. Med. Biol..

[12] Tahrani A.A., Barnett A.H., Bailey C.J. (2016). Pharmacology and therapeutic implications of current drugs for type 2 diabetes mellitus. Nat. Rev. Endocrinol..

[13] Evans J.L., Balkan B., Chuang E., Rushakoff R.J., de Groot L.J., Chrousos G., Dungan K., Feingold K.R., Grossman A., Hershmam J.M., Koch C., Korbonits M., McLachlan R., New M. (2000). Oral and injectable (non-insulin) pharmacological agents for type 2 diabetes. Endotext.

[14] Ginter E., Simko V. (2012). Type 2 diabetes mellitus, pandemic in 21st century. Adv. Exp. Med. Biol..

[15] Andallu B., Radhika B., Suryakantham V. (2003). Effect of aswagandha, ginger and mulberry on hyperglycemia and hyperlipidemia. Plant Foods Hum. Nutr..

[16] Akhani S.P., Vishwakarma S.L., Goyal R.K. (2004). Antidiabetic activity of *Zingiber officinale* in streptozotocin induced type I diabetic rats. J. Pharm. Pharmacol..

[17] Weidner M.S., Sigwart K. (2000). The safety of a ginger extract in the rat. J. Ethnopharmacol..

[18] Gong F., Fung Y.S., Liang Y.Z. (2004). Determination of volatile components in ginger using gas chromatography-mass spectrometry with resolution improved by data processing techniques. J. Agric. Food Chem..

[19] Sekiya K., Ohtani A., Kusano S. (2004). Enhancement of insulin sensitivity in adipocytes by ginger. BioFactors.

[20] Li Y., Tran V.H., Duke C.C., Roufogalis B.D. (2012). Gingerols of *Zingiber officinale* enhance glucose uptake by increasing cell surface GLUT4 in cultured L6 myotubes. Planta Med..

[21] Lee J.O., Kim N., Lee H.J., Moon J.W., Lee S.K., Kim S.J., Kim J.K., Park S.H., Kim H.S. (2015). [6]-Gingerol affects glucose metabolism by dual regulation via the AMPKα2-mediated AS160-Rab5 pathway and AMPK-mediated insulin sensitizing effects. J. Cell. Biochem..

[22] Li Y., Tran V.H., Koolaji N., Duke C., Roufogalis B.D. (2013). (*S*)-[6]-Gingerol enhances glucose uptake in L6 myotubes by activation of AMPK in response to [Ca^2+^]i. J. Pharm. Pharm. Sci..

[23] Son M.J., Miura Y., Yagasaki K. (2015). Mechanisms for antidiabetic effect of gingerol in cultured cells and obese diabetic model mice. Cytotechnology.

[24] Govindarajan V.S. (1982). Ginger-chemistry, technology, and quality evaluation: Part 1. Crit. Rev. Food Sci. Nutr..

[25] Wohlmuth H., Leach D.N., Smith M.K., Myers S.P. (2005). Gingerol content of diploid and tetraploid clones of ginger (*Zingiber officinale* Roscoe). J. Agric. Food Chem..

[26] Chen H., Lv L., Soroka D., Warin R.F., Parks T.A., Hu Y., Zhu Y., Chen X., Sang S. (2012). Metabolism of [6]-shogaol in mice and in cancer cells. Drug Metab. Dispos..

[27] Hwang J.T., Lee M.S., Kim H.J., Sung M.J., Kim H.Y., Kim M.S., Kwon D.Y. (2008). Antiobesity effect of ginsenoside Rg3 involves the AMPK and PPAR-γ signal pathways. Phytother. Res..

[28] Tuncman G., Erbay E., Hom X., de Vivo I., Campos H., Rimm E.B., Hotamisligil G.S. (2006). A genetic variant at the fatty acid-binding protein aP2 locus reduces the risk for hypertriglyceridemia, type 2 diabetes, and cardiovascular disease. Proc. Natl. Acad. Sci. USA.

[29] Valverde A.M., Benito M., Lorenzo M. (2005). The brown adipose cell: A model for understanding the molecular mechanisms of insulin resistance. Acta Physiol. Scand..

[30] Han J.H., Zhou W., Li W., Tuan P.Q., Khoi N.M., Thuong P.T., Na M., Myung C.S. (2015). Pentacyclic triterpenoids from *Astilbe rivularis* that enhance glucose uptake via the activation of Akt and Erk1/2 in C2C12 myotubes. J. Nat. Prod..

[31] Hsieh T.J., Tsai Y.H., Liao M.C., Du Y.C., Lien P.J., Sun C.C., Chang F.R., Wu Y.C. (2012). Anti-diabetic properties of non-polar *Toona sinensis* Roem extract prepared by supercritical-CO_2_ fluid. Food Chem. Toxicol..

[32] Hsieh C.T., Hsieh T.J., El-Shazly M., Chuang D.W., Tsai Y.H., Yen C.T., Wu S.F., Wu Y.C., Chang F.R. (2012). Synthesis of chalcone derivatives as potential anti-diabetic agents. Bioorg. Med. Chem. Lett..

[33] Haratake A., Watase D., Setoguchi S., Terada K., Matsunaga K., Takata J. (2014). Relationship between the acyl chain length of paradol analogues and their antiobesity activity following oral ingestion. J. Agric. Food Chem..

[34] Nie H., Meng L.Z., Zhang H., Zhang J.Y., Yin Z., Huang X.S. (2008). Analysis of anti-platelet aggregation components of Rhizoma Zingiberis using chicken thrombocyte extract and high performance liquid chromatography. Chin. Med. J. (Engl.).

[35] Suekawa M., Ishige A., Yuasa K., Sudo K., Aburada M., Hosoya E.J. (1984). Pharmacological studies on ginger. I. Pharmacological actions of pungent constituents, (6)-gingerol and (6)-shogaol. J. Pharmacobio-dynam..

[36] Pan M.H., Hsieh M.C., Kuo J.M., Lai C.S., Wu H., Sang S., Ho C.T. (2008). 6-Shogaol induces apoptosis in human colorectal carcinoma cells via ROS production, caspase activation, and GADD 153 expression. Mol. Nutr. Food Res..

[37] Isa Y., Miyakawa Y., Yanagisawa M., Goto T., Kang M.S., Kawada T., Morimitsu Y., Kubota K., Tsuda T. (2008). 6-Shogaol and 6-gingerol, the pungent of ginger, inhibit TNF-α mediated downregulation of adiponectin expression via different mechanisms in 3T3-L1 adipocytes. Biochem. Biophys. Res. Commun..

[38] Kubra I.R., Rao L.J. (2012). An impression on current developments in the technology, chemistry, and biological activities of ginger (*Zingiber officinale* Roscoe). Crit. Rev. Food Sci. Nutr..

[39] Hu F.B. (2003). Sedentary lifestyle and risk of obesity and type 2 diabetes. Lipids.

[40] Shah P., Mudaliar S. (2010). Pioglitazone: Side effect and safety profile. Expert Opin. Drug Saf..

[41] Shih H.C., Chern C.Y., Kuo P.C., Wu Y.C., Chan Y.Y., Liao Y.R., Teng C.M., Wu T.S. (2004). Synthesis of analogues of gingerol and shogaol, the active pungent principles from the rhizomes of *Zingiber officinale* and evaluation of their anti-platelet aggregation effects. Int. J. Mol. Sci..

[42] Dagon Y., Avraham Y., Berry E.M. (2006). AMPK activation regulates apoptosis, adipogenesis, and lipolysis by eIF2α in adipocytes. Biochem. Biophys. Res. Commun..

[43] Zhang B.B., Zhou G., Li C. (2009). AMPK: An emerging drug target for diabetes and the metabolic syndrome. Cell Metab..

[44] Brown J.D., Plutzky J. (2007). Peroxisome proliferator-activated receptors as transcriptional nodal points and therapeutic targets. Circulation.

[45] Li Y., Qi Y., Huang T.H., Yamahara J., Roufogalis B.D. (2008). Pomegranate flower: A unique traditional antidiabetic medicine with dual PPAR-α/-γ activator properties. Diabetes Obes. Metab..

[46] Bhattarai S., Tran V.H., Duke C.C. (2001). The stability of gingerol and shogaol in aqueous solutions. J. Pharm. Sci..

[47] Berti L., Kellerer M., Capp E., Haring H.U. (1997). Leptin stimulates glucose transport and glycogen synthesis in C2C12 myotubes: Evidence for a PI3-kinase mediated effect. Diabetologia.

[48] Ishiyama M., Shiga M., Sakamoto K., Mizoguchi M., He P.A. (1993). New sulfonated tetrazolium salt that produces a highly watersoluble formazan dye. Chem. Pharm. Bull..

[49] Srinivasan K., Ramarao P. (2007). Animal models in type 2 diabetes research: An overview. Indian J. Med. Res..

[50] Zhou M., Xu H., Pan L., Wen J., Liao W., Chen K. (2008). Rosiglitazone promotes atherosclerotic plaque stability in fat-fed ApoE-knockout mice. Eur. J. Pharmacol..

[51] Fang Y.P., Chuang C.H., Wu P.C., Huang Y.B., Tzeng C.C., Chen Y.L., Liu Y.T., Tsai Y.H., Tsai M.J. (2016). Amsacrine analog-loaded solid lipid nanoparticle to resolve insolubility for injection delivery: Characterization and pharmacokinetics. Drug Des. Dev. Ther..

