# Supplementary material for: 6-Paradol and 6-Shogaol, the Pungent Compounds of Ginger, Promote Glucose Utilization in Adipocytes and Myotubes, and 6-Paradol Reduces Blood Glucose in High-Fat Diet-Fed Mice
Source: Int J Mol Sci. 2017 Jan 17;18(1):168. doi: 10.3390/ijms18010168 (PMC5297801; doi:10.3390/ijms18010168)
Supplement: Supplementary file 1 [file ijms-18-00168-s001.pdf]

Supplementary Materials: 6-Paradol and 6-Shogaol, the Pungent Compounds of Ginger, Promote Glucose Utilization in Adipocytes and Myotubes and 6-Paradol Reduces Blood Glucose in High-Fat Diet-Fed Mice

Chien-Kei Wei, Yi-Hong Tsa, Michal Korinek, Pei-Hsuan Hung, Mohamed El-Shazly, Yuan-Bin Cheng, Yang-Chang Wu, Tusty-Jiuan Hsieh and Fang-Rong Chang

1. General

All chemical reagents were commercially available unless otherwise indicated. Analytical thin-layer chromatography (TLC) was performed on a Kieselgel 60, F_{254} (0.20 nm, Merck), and visualization was accomplished with UV light at 254 and 354 nm. Column chromatography was carried out using Merck silica gel 60 (230–400 mesh). ^{1}H NMR spectra were recorded on Varian Unity Plus 400 MHz (Varian Inc., Palo Alto, CA, USA) spectrometers using TMS as an internal standard and D-chloroform (Merck) as the solvent. Chemical shifts were reported in ppm (parts per million), and coupling constants (*J*) were expressed in Hz. The relative purity of the synthesized compounds was quantified based on the GC-MS chromatogram (Thermo Finnigan, Trace GC with Polaris Q mass spectrometer, Thermo Electron Corporation, San Jose, CA, USA; GC Column: HP-5MS 30 m × 0.25 mm × 0.25 μm) and peak area integration using the analysis program (Thermo Xcalibur™, Waltham, MA, USA).

2. Synthesis of 6-Shogaol, 8-Shogaol, 10-Shogaol, 6-Paradol, 8-Paradol and 10-Paradol

2.1. Synthesis of 6-Shogaol, 8-Shogaol and 10-Shogaol

Dry tetrahydrofuran (THF) was added to a flask charged with zingerone (1.0 equiv.) under inert atmosphere. The reaction was cooled to −78 °C; potassium tertiary butoxide (*t*BuOK, 2.5 equiv.) was added in a single portion, and the mixture was kept under stirring for 1 h. Aldehydes (1.2 equiv.) with different alkyl side chain lengths were added slowly. The reaction was kept under stirring for another 2 h and finally quenched by water. After extracting the mixture with dichloromethane, the organic part was evaporated under vacuum. The residue was purified by column chromatography using *n*-hexane/ethyl acetate (4:1) to afford the corresponding racemic gingerol. The synthesized gingerol racemate (1.0 equiv.) was dissolved in dry benzene followed by the addition of *p*-toluenesulfonic acid (0.4 equiv.). The mixture was refluxed for 1 h and quenched by water. After extracting with dichloromethane, the organic part was dried under vacuum. The residue was purified by column chromatography using *n*-hexane/ethyl acetate (4:1) to afford the corresponding shogaol. The yields of 6-, 8- and 10-shogaols were 48%, 49% and 46%, respectively. The ^{1}H NMR spectral data were in agreement with the literature data [1,2]. The purity data are as follows: 6-shogaol (>95%), 8-shogaol (>92%) and 10-shogaol (>95%).

2.2. Synthesis of 6-, 8- and 10-Paradols

6-, 8- or 10-Shogaol (100 mg) was dissolved in 5 mL ethyl acetate. To this solution, 10% palladium on carbon (Pd/C, 82 mg) was added, and the mixture was degassed with a hydrogen balloon. The reaction was stirred under hydrogen atmosphere (1 atm) at room temperature for 12 h. After the reaction completion, the solution was filtered through a short pad of silica gel and washed with dichloromethane. The organic solvent was evaporated under vacuum, and the residue was purified by column chromatography with *n*-hexane/ethyl acetate (4:1) to afford the corresponding paradol. The yields of 6-, 8- and 10-paradols were 56%, 54% and 51%, respectively. The ^{1}H NMR spectral data were in agreement with the literature data [3]. The purity data are as follows: 6-paradol (>95%), 8-paradol (>95%) and 10- paradol (>95%).

3. ^{1}H NMR and ^{13}C NMR Data for 6-Shogaol, 8-Shogaol, 10-Shogaol, 6-Paradol, 8-Paradol and 10-Paradol

(E)-1-(4-Hydroxy-3-methoxyphenyl)-dec-4-en-3-one (6-shogaol): ^{1}H NMR δ: 0.89 (3H, t, *J* = 6.8 Hz), 1.30 (4H, m), 1.45 (2H, m), 2.19 (2H, q, *J* = 7.2 Hz), 2.85 (4H, m), 3.87 (3H, s), 5.49 (1H, brs), 6.09 (1H, d, *J* = 15.6 Hz), 6.69 (1H, d, *J* = 8.0 Hz), 6.71 (1H, brs), 6.82 (2H, m); ^{13}C NMR δ: 14.5, 23.0, 28.3, 30.4, 31.9, 33.0, 42.6, 56.4, 111.7, 114.9 121.4, 130.9, 133.8, 144.4, 146.9, 148.5, 200.4.

(E)-1-(4-Hydroxy-3-methoxyphenyl)-dodec-4-en-3-one (8-shogaol): ^{1}H NMR δ: 0.87 (3H, t, *J* = 6.4 Hz), 1.27 (8H, m), 1.43 (2H, m), 2.19 (2H, q, *J* = 7.2 Hz), 2.84 (4H, m), 3.87 (3H, s), 5.48 (1H, brs), 6.08 (1H, d, *J* = 15.6 Hz), 6.68 (1H, d, *J* = 8.0 Hz), 6.71 (1H, brs), 6.81 (2H, m); ^{13}C NMR δ: 14.7, 23.2, 28.7, 29.6, 29.7, 30.4, 32.3, 33.1, 42.6, 56.4, 111.7, 114.9, 121.4, 130.9, 133.8, 144.4, 146.9, 148.5, 200.4.

(E)-1-(4-Hydroxy-3-methoxyphenyl)-tetradec-4-en-3-one (10-shogaol): ^{1}H NMR δ: 0.88 (3H, t, *J* = 6.8 Hz), 1.26 (12H, m), 1.43 (2H, m), 2.19 (2H, q, *J* = 7.2 Hz), 2.85 (4H, m), 3.87 (3H, s), 5.47 (1H, brs), 6.09 (1H, d, *J* = 15.6 Hz), 6.69 (1H, d, *J* = 8.0 Hz), 6.71 (1H, brs), 6.81 (2H, m); ^{13}C NMR δ: 14.7, 23.3, 28.7, 29.8, 29.9, 30.0, 30.1, 30.5, 32.5, 33.1, 42.6, 56.5, 111.7, 114.9, 121.4, 130.9, 133.9, 144.4, 147.0, 148.5, 200.4.

1-(4-Hydroxy-3-methoxyphenyl)-decan-3-one (6-paradol): ^{1}H NMR δ: 0.87 (3H, t, *J* = 6.8 Hz), 1.25 (8H, m), 1.54 (2H, m), 2.37 (2H, t, *J* = 7.6 Hz), 2.69 (2H, t, *J* = 7.6 Hz), 2.82 (2H, t, *J* = 7.6 Hz), 3.87 (3H, s), 5.50 (1H, brs), 6.66 (1H, d, *J* = 8.0 Hz), 6.69 (1H, brs), 6.82 (1H, d, *J* = 8.0 Hz); ^{13}C NMR δ: 14.6, 23.2, 24.4, 29.6, 29.7, 30.1, 32.2, 43.7, 45.2, 56.4, 111.6, 114.9, 121.3, 133.7, 144.4, 146.9, 211.3.

1-(4-Hydroxy-3-methoxyphenyl)-dodecan-3-one (8-paradol): ^{1}H NMR δ: 0.87 (3H, t, *J* = 6.8 Hz), 1.25 (12H, m), 1.54 (2H, m), 2.36 (2H, t, *J* = 7.6 Hz), 2.69 (2H, t, *J* = 7.6 Hz), 2.82 (2H, t, *J* = 7.6 Hz), 3.87 (3H, s), 5.48 (1H, brs), 6.66 (1H, d, *J* = 8.0 Hz), 6.68 (1H, brs), 6.82 (1H, d, *J* = 8.0 Hz); ^{13}C NMR δ: 14.7, 23.2, 24.4, 29.8, 29.8, 30.0, 30.0, 30.1, 32.4, 43.7, 45.2, 56.5, 111.6, 114.9, 121.3, 133.7, 144.4, 146.9, 211.3.

1-(4-Hydroxy-3-methoxyphenyl)-tetradecan-3-one (10-paradol): ^{1}H NMR δ: 0.88 (3H, t, *J* = 6.8 Hz), 1.24 (16H, m), 1.54 (2H, m), 2.36 (2H, t, *J* = 7.6 Hz), 2.69 (2H, t, *J* = 7.6 Hz), 2.82 (2H, t, *J* = 7.6 Hz), 3.87 (3H, s), 5.48 (1H, brs), 6.66 (1H, d, *J* = 8.0 Hz), 6.68 (1H, brs), 6.82 (1H, d, *J* = 7.6 Hz); ^{13}C NMR δ: 14.7, 23.3, 24.4, 29.8, 29.9, 30.0, 30.1, 30.1, 30.2 (2C), 32.5, 43.7, 45.2, 56.5, 111.6, 114.9, 121.3, 133.7, 144.4, 146.9, 211.3.

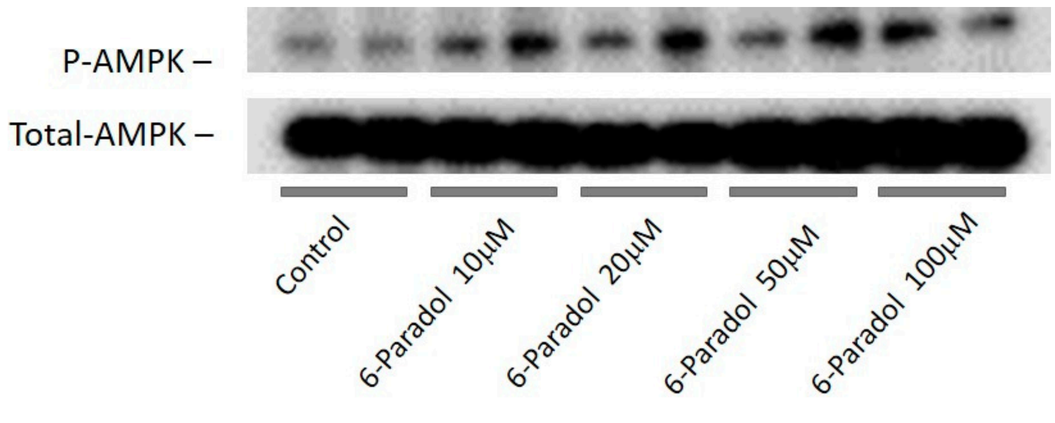

Figure S1. Immunoblotting analysis of different doses of 6-paradol on phosphorylation of AMPK. 3T3-L1 cells were treated with 10–100 μM of 6-paradol for 24 h and immunoblotted in duplicates with phospho-AMPK and total-AMPK antibodies for the detection of AMPK activation.

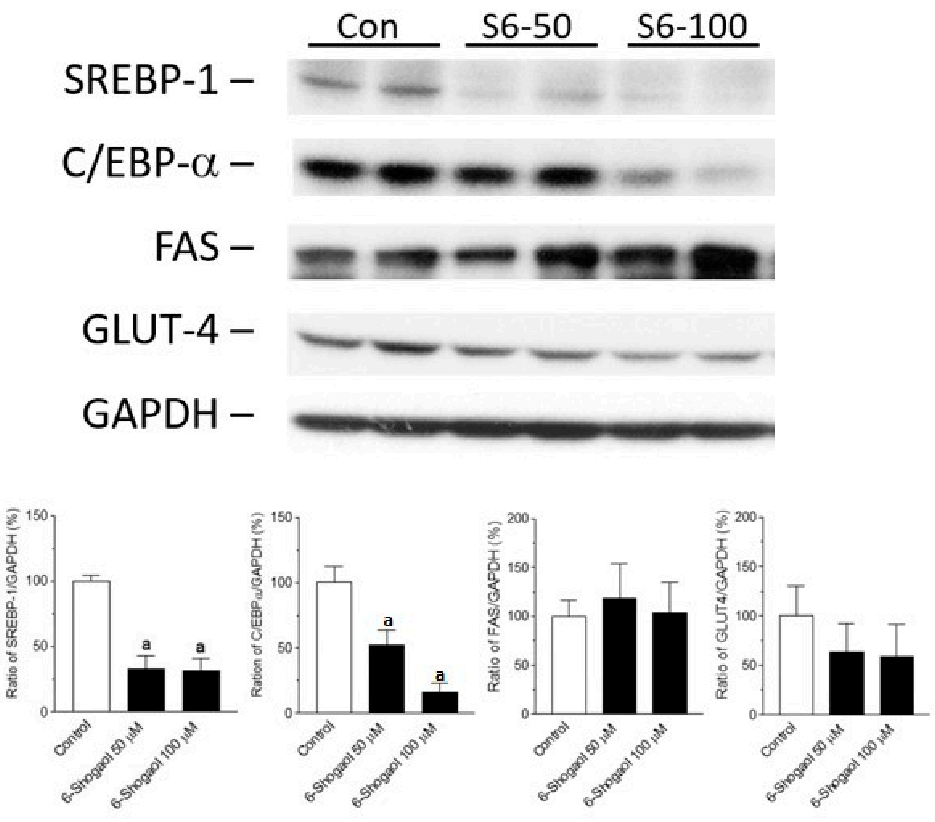

Figure S2. Immunoblotting analysis of protein expression on SREBP-1, C/EBP-α, FAS and GLUT4. 3T3-L1 cells were treated with 50 μM and 100 μM of 6-shogaol for 24 h and immunoblotted in duplicates with SREBP-1, C/EBP-α, FAS, GLUT4 and GAPDH antibodies for the detection of SREBP-1, C/EBP-α, FAS and GLUT4 protein expression. Results are the mean ± SEM of three independent experiments. [a] $p < 0.001$ indicates a significant difference compared with the control group.

References

1. Sang, S.; Hong, J.; Wu, H.; Liu, J.; Yang, C.S.; Pan, M.H.; Badmaev, V.; Ho, C.T. Increased growth inhibitory effects on human cancer cells and anti-inflammatory potency of shogaols from *Zingiber officinale* relative to gingerols. *J. Agric. Food Chem.* **2009**, *22*, 10645–10650.
2. Eton, H.; Kondon, T.; Noda, R.; Singh, I.P.; Sekiwa, Y.; Morimitsu, K.; Kubota, K. Shogaols from *Zingiber officinale* as promising antifouling agents. *Biosci. Biotechnol. Biochem.* **2002**, *66*, 1748–1750.
3. Groblacher, B.; Maier, V.; Kunert, O.; Bucar, F. Putative mycobacterial efflux inhibitors from the seeds of *Aframomum melegueta*. *J. Nat. Prod.* **2012**, *75*, 1393–1399.
